# Supplementary material for: Novel Strategy for Non-Aqueous Bioconjugation of Substituted Phenyl-1,2,4-triazole-3,5-dione Analogues
Source: Molecules. 2022 Oct 7;27(19):6667. doi: 10.3390/molecules27196667 (PMC9570748; doi:10.3390/molecules27196667)
Supplement: Supplementary file 1 [file molecules-27-06667-s001.zip › molecules-1929249-supplementary.pdf]

Supplementary Information

Novel strategy for non-aqueous bioconjugation of substituted Phenyl-1,2,4-triazole-3,5-dione analogues

Hugh G. Hiscocks [1], Alison T. Ung [1,] and Giancarlo Pascali [2,*]

[1]School of Mathematical and Physical Sciences, Faculty of Science, University of Technology Sydney, Broadway, NSW 2007, Australia

[2]School of Chemistry, University of New South Wales, Kensington, NSW 2052, Australia

[3]Prince of Wales Hospital, Nuclear Medicine and PET, Randwick, NSW 2031, Australia

[4]National Imaging Facility, University of New South Wales, Kensington, NSW 2052, Australia

*Correspondence: G.pascali@unsw.au

Table of contents Page

Table S1 2
Table S2 2
General procedure for the synthesis of N-({phenyl carbamoyl}amino)ethoxyformamide derivatives (**3a-f**) 2
Spectral data of compounds **3a-f** 2-4
General procedure for synthesis of phenyl-1,2,4-triazolidine-3,5-dione's derivatives (4a-f) 4
Spectral data of compounds **4a-f** 4-6
Chromatograms Figures S1-S6 6-8
Table S3 and Figure S7 9
NMR spectra of new compounds **6a-f** 10-24

Table S1: Isolated yields of N-({phenyl carbamoyl}amino)ethoxyformamide derivatives (**3a-f**)

3	R =	Yield (%)
**a**	SF_5	88
**b**	CF_3	78
**c**	F	79
**d**	H	81
**e**	Me	51
**f**	OMe	43

Table S2: Isolated yields of N-phenyl-1,2,4-triazolidine-3,5-dione derivatives (**4a-f**)

4	R =	Yield (%)
**a**	SF_5	90
**b**	CF_3	82
**c**	F	65
**d**	H	78
**e**	Me	73
**f**	OMe	67

General procedure for the synthesis of N-({phenyl carbamoyl}amino)ethoxyformamide derivatives (3a-f):

1,1'-Carbonyldiimidazole (450 mg, 2.765 mmol) was suspended in anhydrous dichloromethane (10 mL) under nitrogen. The para substituted aniline (2 mmol) was added and the reaction mixture was refluxed at 50°C for 18 hr. Ethyl hydrazinecarboxylate (209 mg; 2 mmol) dropwise to the solution and the reaction was stirred at 50°C for 45 min and a further 2 hr under nitrogen flow. The precipitate was collected by vacuum filtration and washed with cold dichloromethane, the crude product was further dried *in vacuo*

*N-({[4-(pentafluoro-λ⁶-sulfanyl)phenyl]carbamoyl}amino)ethoxyformamide (**3a**)*

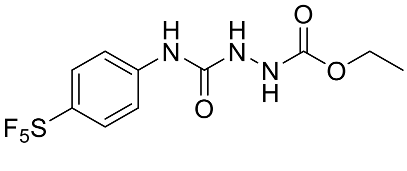

Compound **3a** was obtained as a white solid (620 mg, 1.78 mmol, 88%). Calcd for $C_{10}H_{13}F_5N_3O_3S$ $[M+H]^+$ 350.0597, $[M+H]^+$ Found 350.0592; **^{1}H-NMR (DMSO**): δ 7.75 (d, J = 9.5 Hz, 2H), 7.65 (d, J = 8.5 Hz , 2H), 4.05 (q, J = 7, 14 Hz), 1.2 (t, J = 6.5 Hz) **^{13}C-NMR (DMSO):** 157.05, 155.50, 146.22, 146.06, 143.43, 126.79, 117.98, 60.80, 14.71; **^{19}F-NMR (DMSO):** δ 89.39 (q, J = 150.62, 1F), 64.20 (d, J = 150.62, Hz, 4F); **IR (KBr):** 3315 (w), 1703 (w), 1673 (w), 1565 (m), 1507 (w), 1237 (w), 1105 (w), 840 (m), 810 (s) cm^{-1}

*N-({[4-(trifluoromethyl)phenyl]carbamoyl}amino)ethoxyformamide (**3b**)*

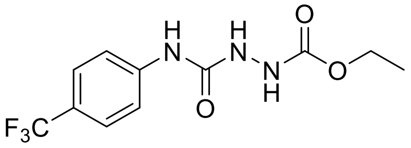

Compound **3b** was obtained as a white solid (462 mg, 1.59 mmol, 78%). **HRMS (EI)** Calcd for $C_{11}H_{13}F_3N_3O_3$ $[M+H]^+$ 292.0909, $[M+H]^+$ Found 292.0903; **^{1}H-NMR (DMSO)**: δ 7.67 (br s, 2H), 7.59 (d, 2H, J = 10 Hz); **^{13}C-NMR (DMSO):** δ 156.91, 155.40, 143.55, 128.55, 125.92, 125.88, 123.23, 122.29, 121.98, 121.66, 121.34, 120.54, 118.15, 60.60, 14.53; **^{19}F-NMR (DMSO):** δ -60.13 (s); **IR (KBr):** 3302 (w), 1701 (w), 1671 (w), 1612 (w), 1559 (w), 1509 (w), 1410 (w), 1325 (m), 1231 (m), 1159 (m), 1108 (s), 1067 (s), 1012 (w), 844 (s) cm^{-1}

*N-({[4-fluorophenyl]carbamoyl}amino)ethoxyformamide (**3c**)*

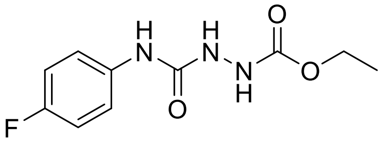

Compound **3c** was obtained as a white solid (382 mg, 1.59 mmol, 79%). **HRMS (EI)** Calcd for $C_{10}H_{13}FN_3O_3$ $[M+H]^+$ 242.0940, $[M+H]^+$ Found 242.0938; **^{1}H-NMR (DMSO)**: δ 7.47 (d, J = 3.5 Hz , 2H); 7.08 (d, J = 8.5 Hz, 2H), 4.06 (q, J = 7, 14 Hz, 2H), 1.19 (t, J = 7 Hz, 3H); **^{13}C-NMR (DMSO):** δ 158.48, 156.92, 156.13, 155.68, 152.70, 136.03, 136.01, 135.97, 120.23, 120.02, 119.94, 115.34, 115.12, 114.91, 60.49, 14.52; **^{19}F-NMR (DMSO):** δ -121.61; **IR (KBr):** 3274 (s), 2983 (w), 1735 (s) 1682 (s) 1559 (s), 1505 (s), 1410 (w), 1320 (w), 1233 (s), 1207 (s), 1053 (w), 1023 (w), 848 (s), 788 (m) cm^{-1}

*N-({phenyl carbamoyl}amino)ethoxyformamide (**3d**)*

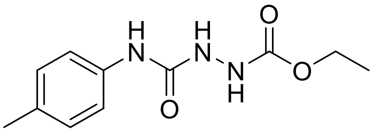

Compound **3d** was obtained as a tan brown solid (362 mg, 1.62 mmol, 81%). **HRMS (EI)** Calcd for $C_{10}H_{14}N_3O_3$ $[M+H]^+$ 224.1035, $[M+H]^+$ Found 224.1031 **^{1}H-NMR (DMSO)**: δ 7.45 (d, J = 7.5 Hz, 2H), 7.24 (t, 7.5 Hz, 2H), 6.94 (t, J = 7.5 Hz, 2H), 4.06 (q, 7, 14 Hz, 3H), 1.19 (t, J = 6.5 Hz, 3H); **^{13}C-NMR (DMSO):** δ 157.13, 155.75, 139.86, 128.95, 128.76, 121.97, 118.62, 118.43, 60.67, 14.72; **IR (KBr):** 3300 (m), 3259 (m), 2980 (s), 1744 (s), 1595 (s), 1554 (s), 1442 (m), 1317 (w), 1226 (s), 1202 (s), 1053 (m), 741 (s), 695 (s) cm^{-1}

*N-({[4-methylphenyl]carbamoyl}amino)ethoxyformamide (**3e**)*

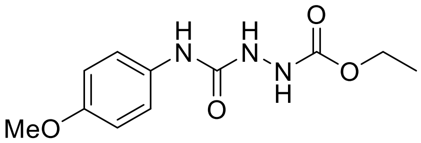

Compound **3e** was obtained as a white solid (245 mg, 1.03 mmol, 51%). **HRMS (EI)** Calcd for $C_{11}H_{16}N_3O_3$ $[M+H]^+$ 238.1191, $[M+H]^+$ Found 238.1192; **^{1}H-NMR (DMSO)**: δ 7.33 (d, J = 8 Hz, 2H), 7.04 (d, J = 8 Hz, 2H), 4.05 (q, J = 7.5, 14.5 Hz, 2H), 2.22 (s, 3H), 1.19 (t, J = 6.5 Hz, 3H); **^{13}C-NMR (DMSO):** δ 157.15, 155.78, 137.27, 130.75, 129.15, 118.74, 60.65, 20.48, 14.73; **IR (KBr):**

3315 (m), 3267 (m), 2987 (w), 2905 (w), 1742 (s), 1682 (m), 1640 (m), 1597 (s), 1550 (s), 1515 (s) 1304 (m), 1202 (s), 825 (s), 663 (s) cm^{-1}

*N-({[4-methoxyphenyl]carbamoyl}amino)ethoxyformamide (**3f**)*

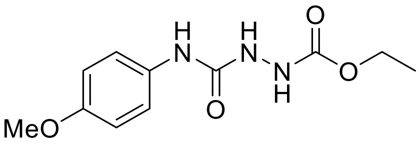

Compound **3f** was obtained as a white solid (219 mg, 0.86 mmol, 43%). **HRMS (EI)** Calcd for $C_{11}H_{16}N_3O_4$ $[M+H]^+$ 254.1140, $[M+H]^+$ Found 254.1131; **^{1}H-NMR (DMSO)**: δ 7.35 (d, J = 9 Hz, 2H), 6.83 (d, J = 7 Hz, 2H), 4.04 (q, J = 10, 20 Hz, 2H), 3.70 (s, 3H), 1.19 (t, J = 6.5 Hz, 3H); **^{13}C-NMR (DMSO):** 156.98, 155.80, 154.41, 132.72, 120.72, 120.28, 113.78, 60.47, 55.13, 14.55; **IR (KBr):** 3278 (m), 2978 (m), 1736 (s), 1682 (m), 1640 (m), 1597 (s), 1556 (s), 1513 (s), 1455 (w), 1295 (m) 1231 (s), 1187 (s), 834 (s), 756 (m), 665 (m) cm^{-1}

General procedure for synthesis of phenyl-1,2,4-triazolidine-3,5-dione's derivatives (4a-f)

A 100mL RBF is charged 4M potassium hydroxide (10mL) and *N*-({[4-(pentafluoro-λ^6-sulfanyl)phenyl]carbamoyl}amino)ethoxyformamide **(**517 mg, 1.5 mmol). The suspension was refluxed at 100°C until the solid had completely dissolved, at which point the reaction was left for an additional hour and the solution was filtered. Upon cooling to room temperature, the solution was acidified *via* the dropwise addition of concentrated hydrochloric acid. The precipitate was isolated *via* vacuum filtration and the mother liquor was concentrated *in vaccuo*. The subsequent residue was extracted twice with hot ethanol (2x10 mL) and the combined organic layers were then filtered, concentrated *in vaccuo* and lyophilised.

*4-[4-(pentafluoro-λ^6-sulfanyl)phenyl]-1,2,4-triazolidine-3,5-dione (**4a**)*

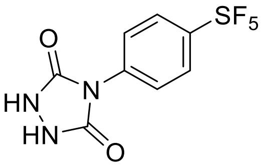

Compound **4a** was obtained as a white solid (410 mg, 1.34 mmol, 90%). **HRMS (EI)** Calcd for $C_8H_7N_3O_2SF_5$ $[M+H]^+$ 304.0179, $[M+H]^+$ Found 304.0178; **^{1}H-NMR (DMSO)**: δ 8.04 (d, J = 7.5 Hz, 2H), 7.81 (d, J = 9 Hz, 2H); **^{13}C-NMR (DMSO):** δ 152.58, 150.92, 150.76, 135.67, 126.75, 126.71, 125.79; **^{19}F-NMR (DMSO):** δ 86.95 (q, J = 86.48, 1F), 64.27 (d, J = 150.4 Hz, 4F); **IR (KBr):** 3052 (w), 1697 (m), 1507 (w), 1220 (w), 1107 (m), 834 (s), 747 (s)

*4-[4-(trifluoromethyl)phenyl]-1,2,4-triazolidine-3,5-dione (**4b**)*

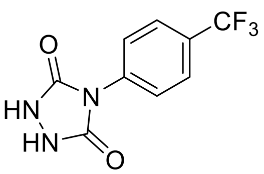

Compound **4d** was obtained as a pale yellow solid (304 mg, 1.24 mmol, 82%). **HRMS (EI)** Calcd for $C_9H_7F_3N_3O_2$ $[M+H]^+$ 246.0490, $[M+H]^+$ Found 246.0496; **^{1}H-NMR (DMSO)**: δ 7.86 (d, 2H, J = 10 Hz), 7.77 (d, 2H, J = 10 Hz); **^{13}C-NMR (DMSO):** δ 152.77, 135.92, 127.82, 126.07, 125.98,

122.82; **^{19}F-NMR (DMSO):** δ 60.96 (s, 1F) ; **IR (KBr):** 3063 (w), 1697 (s, br), 1615 (w), 1420 (s), 1319 (s) 1174 (s), 1120 (s), 1069 (m), 1021 (w), 848 (m), 790 (w), 762 (m) cm^{-1}

*4-[4-(fluoro)phenyl]-1,2,4-triazolidine-3,5-dione (**4c**)*

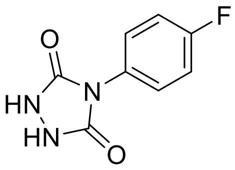

Compound **4c** was obtained as a white solid (190 mg, 0.97 mmol, 65%). **HRMS (EI)** Calcd for $C_8H_7FN_3O_2$ [M+H]$^+$ 196.0522, [M+H]$^+$ Found 196.0527; **^{1}H-NMR (DMSO**): δ 7.49 (br s, 2H), 7.31 (t, J = 10.5, 20.5 Hz, 2H); **^{13}C-NMR (DMSO):** δ 162.23, 159.91, 153.46, 128.40, 115.98; **^{19}F-NMR (DMSO):** δ -114.04; **IR (KBr):** 3207 (w), 3160 (w), 3088 (w), 1682 (s), 1602 (w), 1511 (s), 1436 (m), 1215 (s), 1155 (w), 1116 (w), 1086 (w), 844 (s), 790 (s), 758 (s) cm^{-1}

*4-phenyl-1,2,4-triazolidine-3,5-dione (**4d**)*

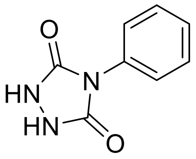

Compound **4d** was obtained as a brown crystalline solid (133 mg, 0.78 mmol, 78%). **HRMS (EI)** Calcd for $C_8H_8N_3O_2$ [M+H]$^+$ 178.0617, [M+H]$^+$ Found 178.0614; **^{1}H-NMR (DMSO**): δ 7.50-7.36 (5H); **^{13}C-NMR (DMSO):** δ 153.53, 132.09, 128.97, 127.78, 126.24; **IR (KBr):** 3151 (w), 3103 (w) 3054 (w), 1777 (s, br), 1679 (w), 1597 (m), 1504 (m), 1436 (w), 1220 (s), 1177 (s), 1116 (m), 911 (w), 892 (m), 756 (m), 689 (s, br) cm^{-1}.

*4-[4-(methyl)phenyl]-1,2,4-triazolidine-3,5-dione (**4e**)*

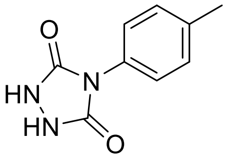

Compound **4e** was obtained as a white crystalline solid (209 mg, 1.1 mmol, 73%). **HRMS (EI)** Calcd for $C_9H_{10}N_3O_2$ [M+H]$^+$ 192.0773, [M+H]$^+$ Found 192.0773; **^{1}H-NMR (DMSO**): δ 7.31 (d, 8 Hz, 2H), 7.27 (d, 8.5 Hz, 2H), 2.34 (s, 3H); **^{13}C-NMR (DMSO):** δ 153.75, 137.30, 129.47, 129.44, 126.14, 20.86; **IR (KBr):** 3097 (w), 3170 (w), 3062 (w), 1697 (s), 1615 (w), 1522 (w), 1420 (br m), 1321 (s), 1174 (s), 1120 (s), 848 (m), 792 (m), 762 (m) cm^{-1}

*4-[4-(methoxy)phenyl]-1,2,4-triazolidine-3,5-dione (**4f**)*

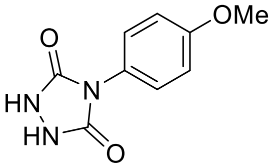

Compound **4f** was obtained as a brown crystalline solid (208 mg, 1 mmol, 67%). **HRMS (EI)** Calcd for $C_9H_9N_3O_3$ $[M+H]^+$ 208.0722, $[M+H]^+$ Found 208.0726; **^{1}H-NMR (DMSO)**: δ 6.52 (d, J = 10 Hz, 2H), 6.22 (d, J = 10 Hz, 2H), 3.02 (s, 3H); **^{13}C-NMR (DMSO):** δ 158.49, 153.75, 127.64, 124.48, 114.04, 53.37; **IR (KBr):** 3227 (br s), 1766 (w), 1673 (br s), 1608 (m), 1507 (s), 1444 (m), 1241 (m), 1215 (w), 1170 (w), 1116 (w), 834 (s), 786 (s), 762 (s) cm^{-1}.

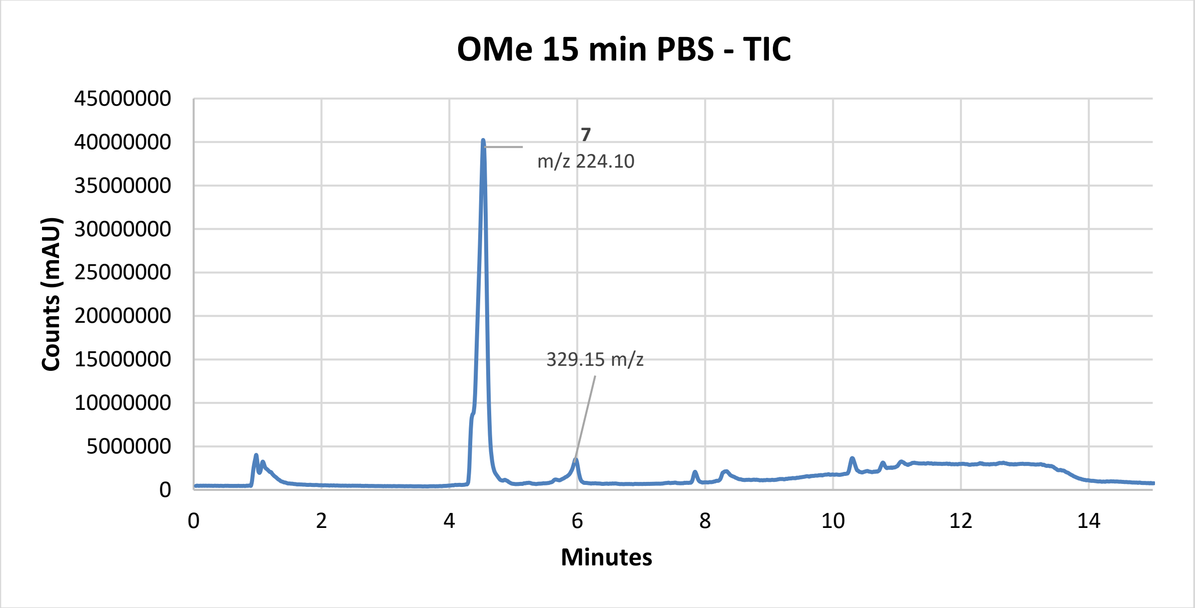

Figure S1. TIC chromatogram of **6f** after 15 minutes reaction time in phosphate-buffered saline, representative of the reaction being performed under aqueous conditions as previously reported in the literature

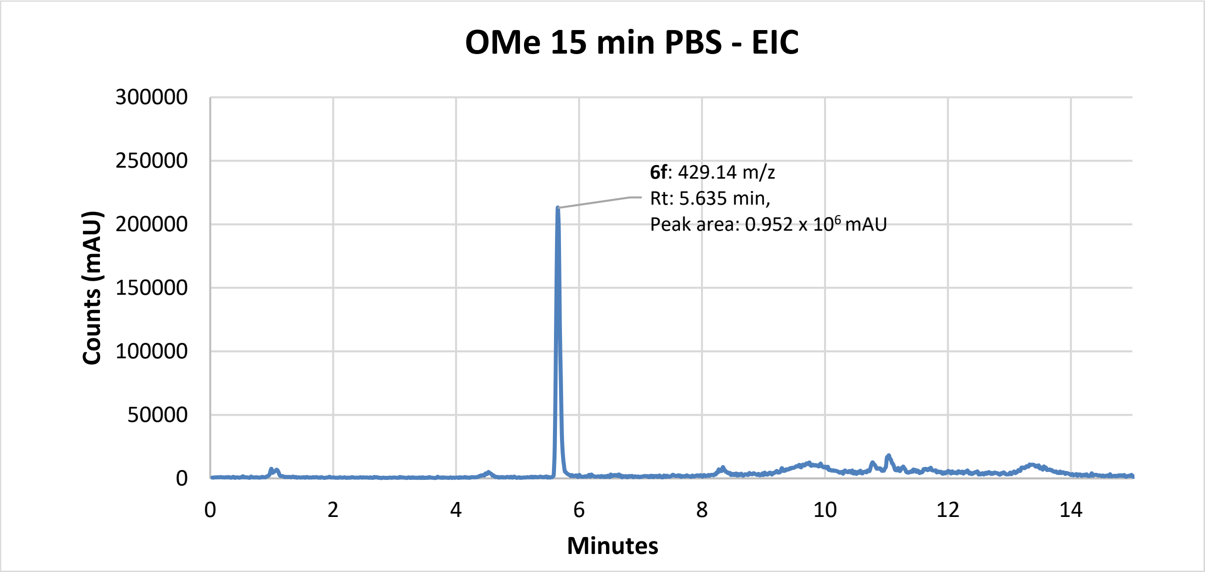

Figure S2. EIC chromatogram of **6f** after 15 minutes reaction time in phosphate-buffered saline, representative of the reaction being performed under aqueous conditions as previously reported in the literature with relatively low conversion

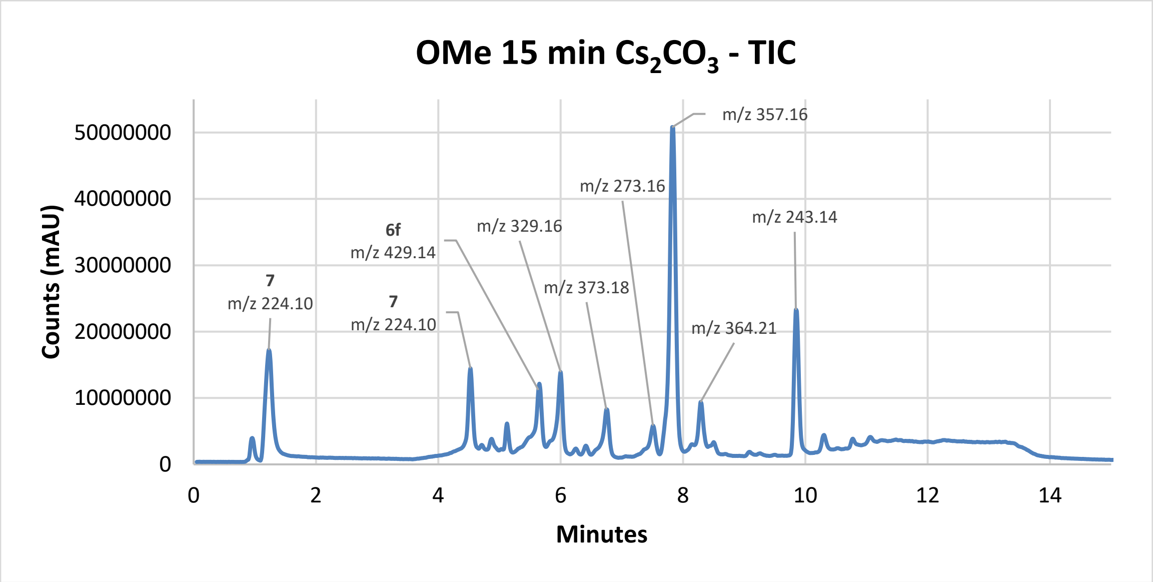

Figure S3. TIC chromatogram of **6f** after 15 minutes reaction time in acetonitrile and caesium carbonate (2 equiv.), representative of synthetic scale reaction performed under Condition **A**

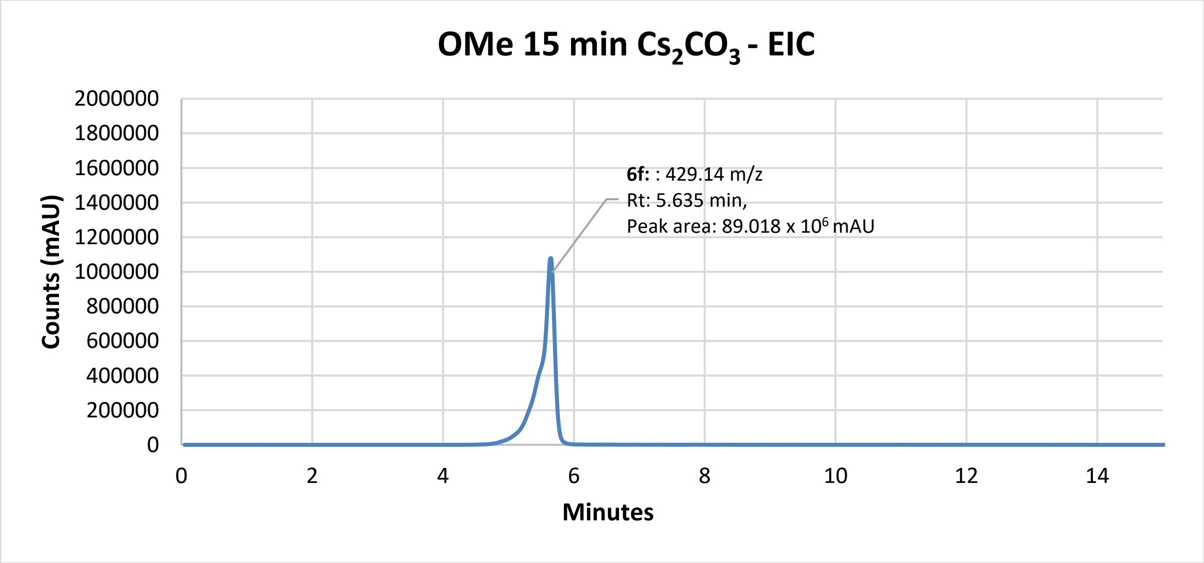

Figure S4. TIC chromatogram of **6f** after 15 minutes reaction time in acetonitrile and caesium carbonate (2 equiv.), representative of synthetic scale reaction performed under Condition **A.** Despite obtaining the highest mAU reading in the LC-MS catalytic study, this reaction gave numerous by-products and >5% **6f** on a synthetic scale.

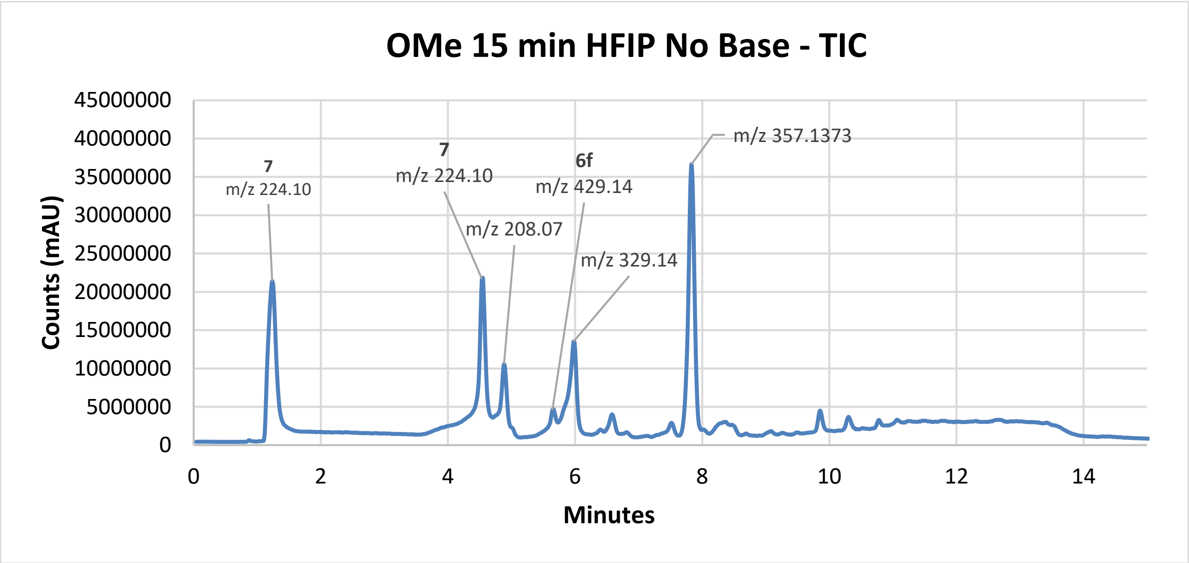

Figure S5. TIC chromatogram of **6f** after 15 minutes reaction time in acetonitrile and HFIP (2 equiv.), representative of synthetic scale reaction performed under Condition **A.** Despite obtaining the highest mAU reading in the LC-MS catalytic study, this reaction proceeded slowly on a synthetic scale.

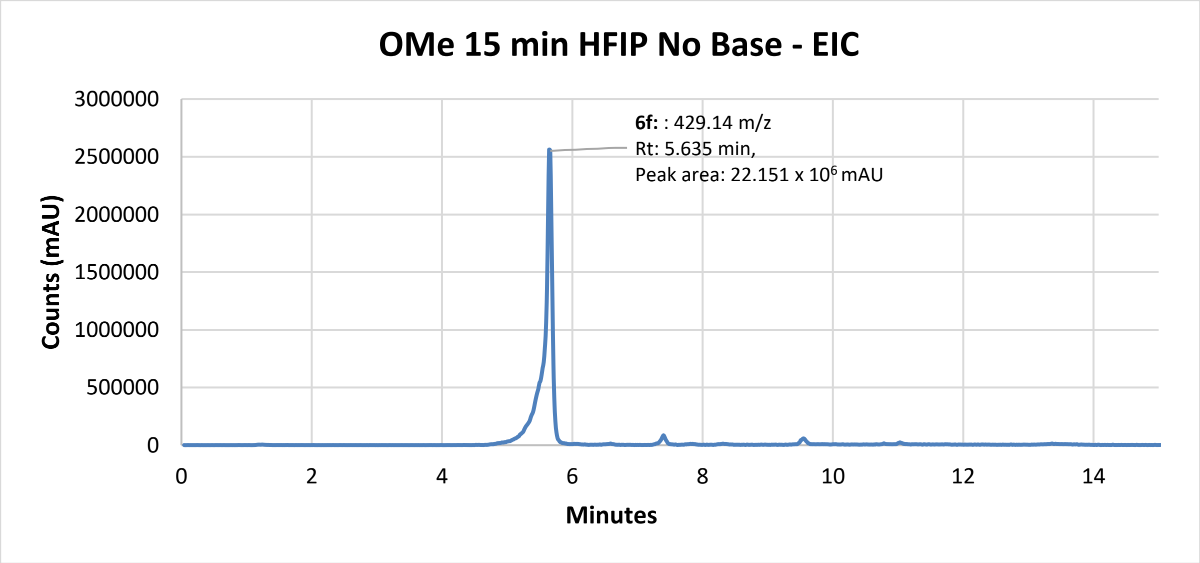

Figure S6. EIC chromatogram of **6f** after 15 minutes reaction time in acetonitrile and HFIP (2 equiv.), representative of synthetic scale reaction performed under Condition **A.** Despite obtaining a relatively high mAU reading in the LC-MS catalytic study, this reaction proceeded slowly on a synthetic scale.

Table S3: The three conditions tested on a synthetic scale based on results obtained from LC-MS catalytic experiments. Condition A: **5a** decomposed rapidly to give numerous by-products and < 5% yield of **6a**, Condition B: Reaction was very slow (> 12 hrs), rendering it unsuitable for the purposes of bioconjugation, Condition C: Reaction proceeded rapidly with minimal by-product formation.

Reaction (6a)	Conditions	Yield (%)
**A**	MeCN, Cs_2CO_3 (2 equiv.)	< 5
**B**	MeCN, HFIP (2 equiv.)	No reaction
**C**	DCM, HFIP (2 equiv.)	54

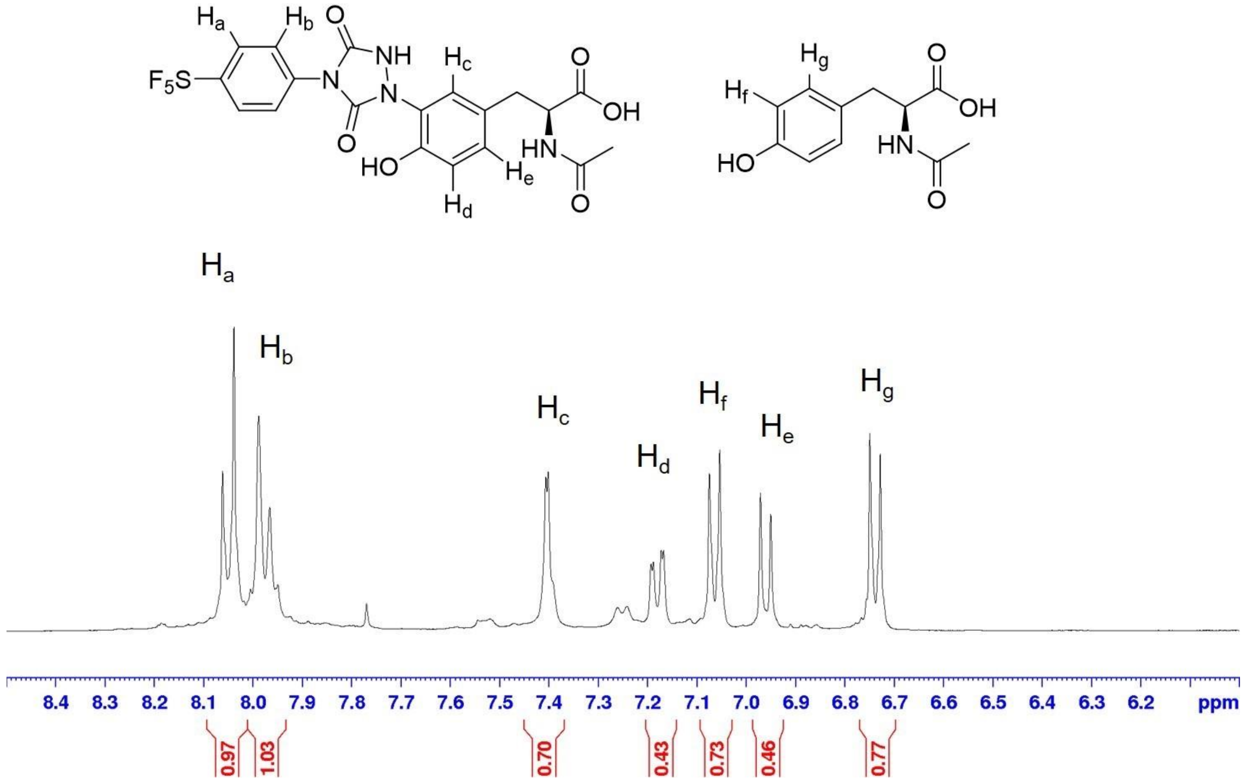

Figure S7: Example ^{1}H-NMR spectra of the crude product **6a** used for calculation of molar conversion using relative integration of aromatic peaks (H_e/H_g)

CF3 PTAD Conj. - 20% HFIP/DCM

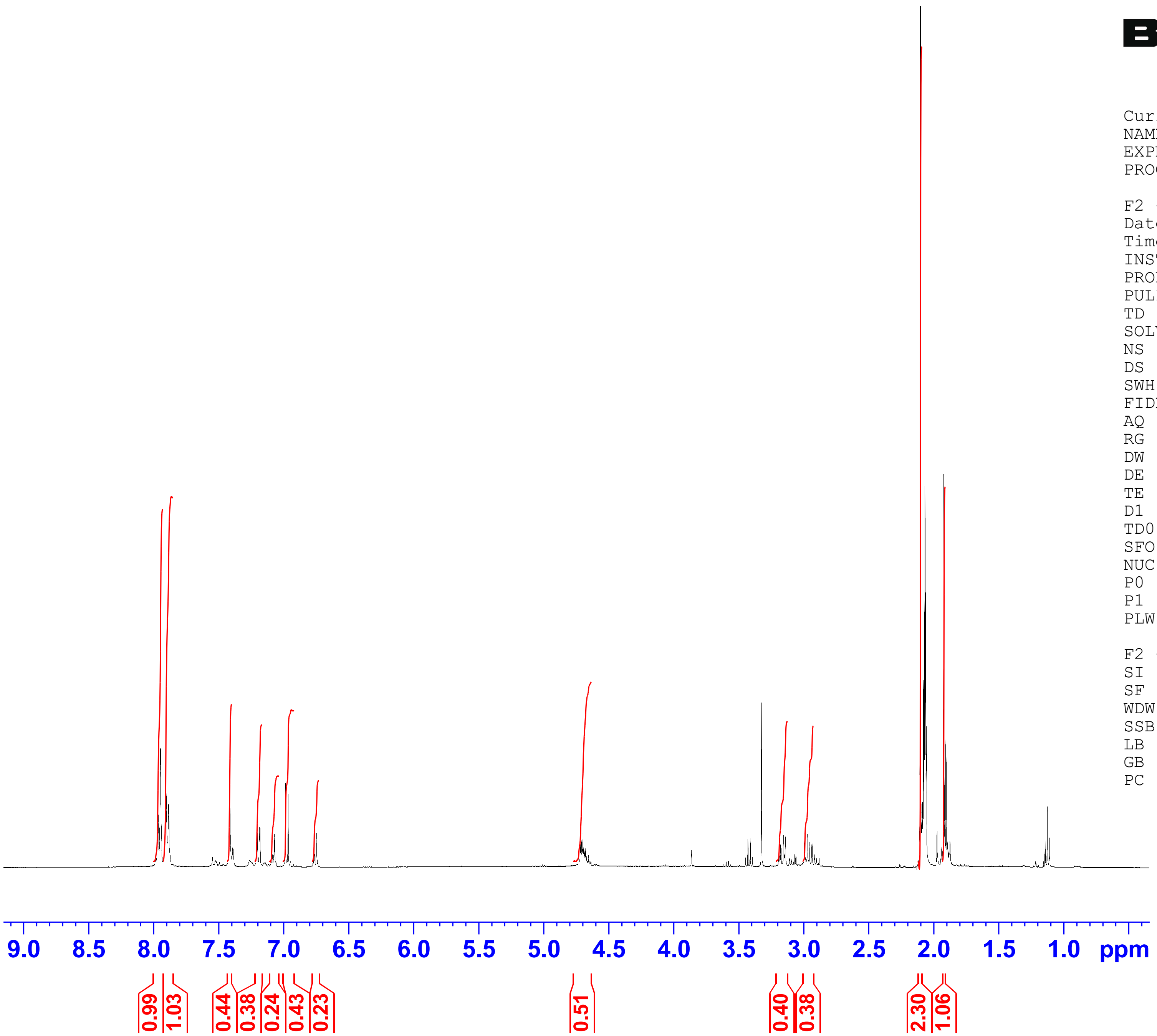

Current Data Parameters
NAME Jul04-2022
EXPNO 15
PROCNO 1

F2 - Acquisition Parameters
Date_ 20220705
Time 9.30 h
INSTRUM AVNEO
PROBHD Z175272_0008 (
PULPROG zg30
TD 131072
SOLVENT Acetone
NS 32
DS 2
SWH 8196.722 Hz
FIDRES 0.125072 Hz
AQ 7.9953918 sec
RG 101
DW 61.000 usec
DE 13.54 usec
TE 298.0 K
D1 1.00000000 sec
TD0 1
SFO1 400.1524709 MHz
NUC1 1H
P0 3.33 usec
P1 10.00 usec
PLW1 19.63299942 W

F2 - Processing parameters
SI 65536
SF 400.1500000 MHz
WDW EM
SSB 0
LB 0.30 Hz
GB 0
PC 1.00

Figure S8. The ^1H NMR (400 MHz, $(CD_3)_2CO$) of compound **6a**

SF5 PTAD Conj.

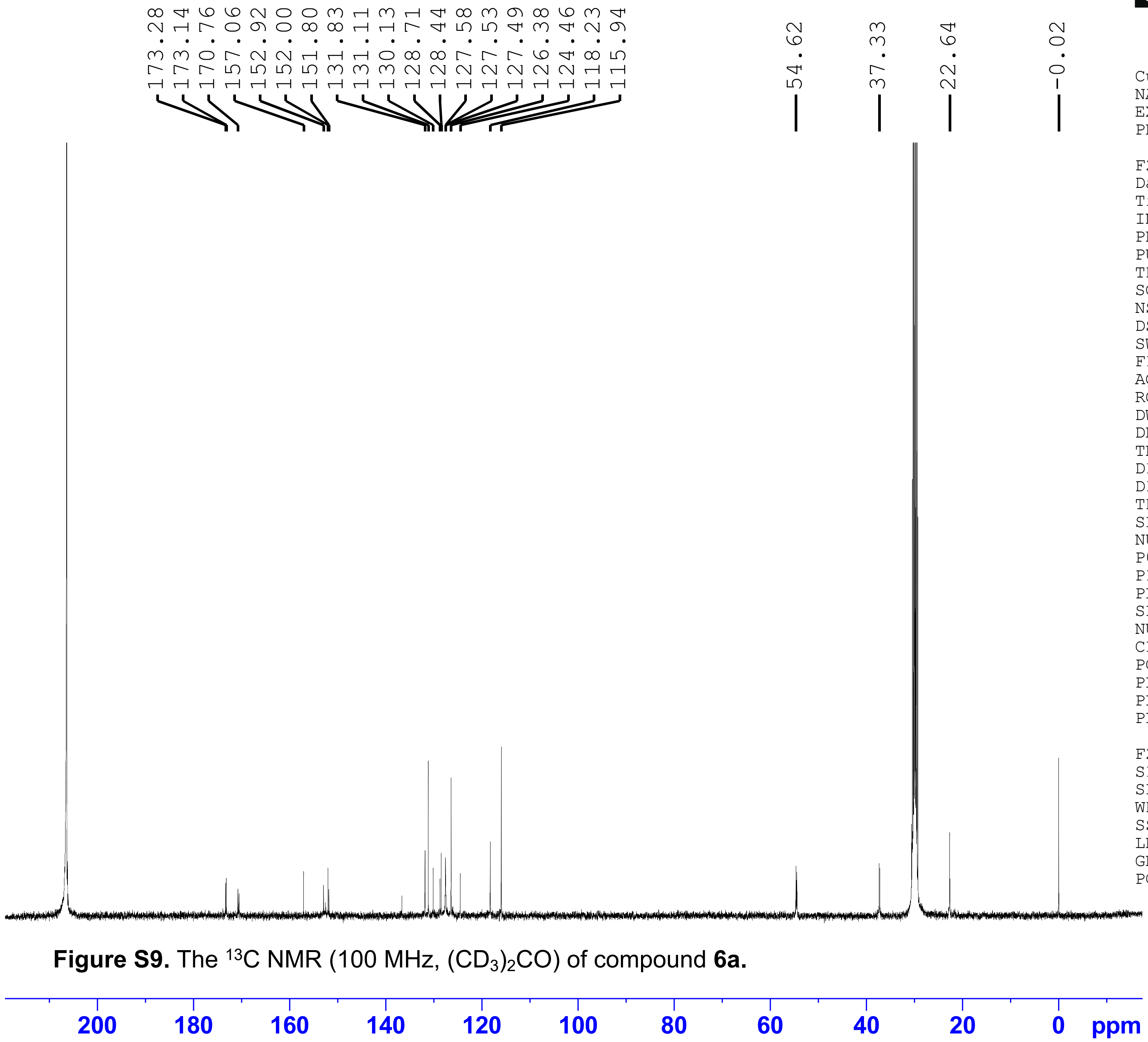

Figure S9. The ^{13}C NMR (100 MHz, $(CD_3)_2CO$) of compound **6a.**

```
Current Data Parameters
NAME      SF5 PTAD Conj - 13C
EXPNO                   1
PROCNO                  1

F2 - Acquisition Parameters
Date_            20220822
Time                23.38 h
INSTRUM             AVNEO
PROBHD   Z175272_0008 (
PULPROG            zgpg30
TD                  65536
SOLVENT           Acetone
NS                   3000
DS                      4
SWH             23809.523 Hz
FIDRES           0.726609 Hz
AQ              1.3762560 sec
RG                    101
DW                 21.000 usec
DE                   6.50 usec
TE                  298.0 K
D1             2.00000000 sec
D11            0.03000000 sec
TD0                     1
SFO1          100.6278593 MHz
NUC1                  13C
P0                   3.33 usec
P1                  10.00 usec
PLW1          58.38199997 W
SFO2          400.1516006 MHz
NUC2                   1H
CPDPRG[2          waltz65
PCPD2               90.00 usec
PLW2          19.63299942 W
PLW12          0.24237999 W
PLW13          0.12192000 W

F2 - Processing parameters
SI                  32768
SF            100.6177093 MHz
WDW                    EM
SSB                     0
LB                   1.00 Hz
GB                      0
PC                   1.40
```

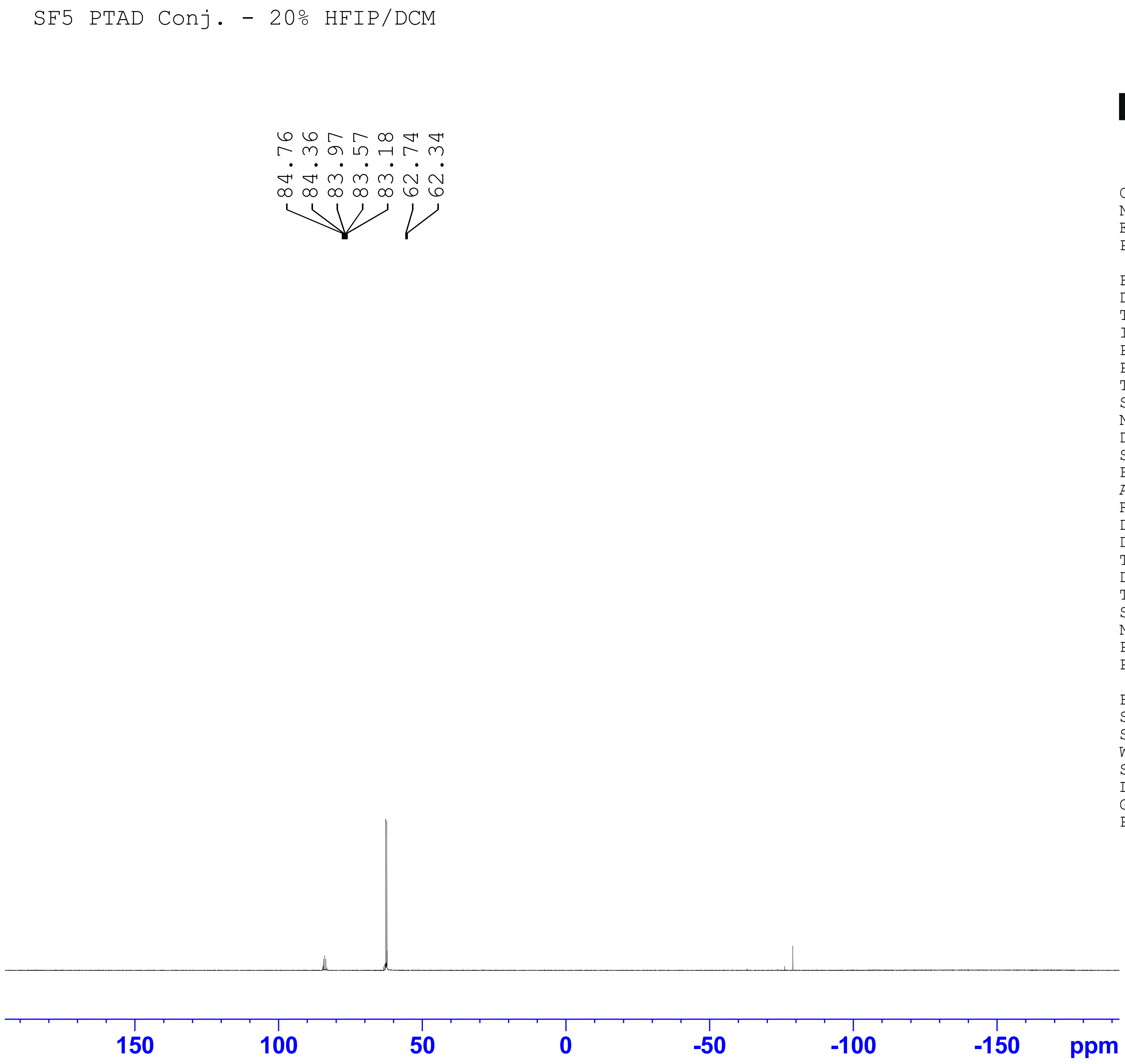

Current Data Parameters
NAME Jul04-2022
EXPNO 20
PROCNO 1

F2 - Acquisition Parameters
Date_ 20220707
Time 1.38 h
INSTRUM AVNEO
PROBHD Z175272_0008 (
PULPROG zg
TD 131072
SOLVENT Acetone
NS 128
DS 4
SWH 147058.828 Hz
FIDRES 2.243940 Hz
AQ 0.4456448 sec
RG 101
DW 3.400 usec
DE 6.50 usec
TE 298.0 K
D1 1.00000000 sec
TD0 1
SFO1 376.5171850 MHz
NUC1 19F
P1 12.00 usec
PLW1 45.00000000 W

F2 - Processing parameters
SI 65536
SF 376.5171850 MHz
WDW EM
SSB 0
LB 0.30 Hz
GB 0
PC 1.00

Figure S10. The ^{19}F NMR (376.5 MHz, $(CD_3)_2CO$) of compound **6a**.

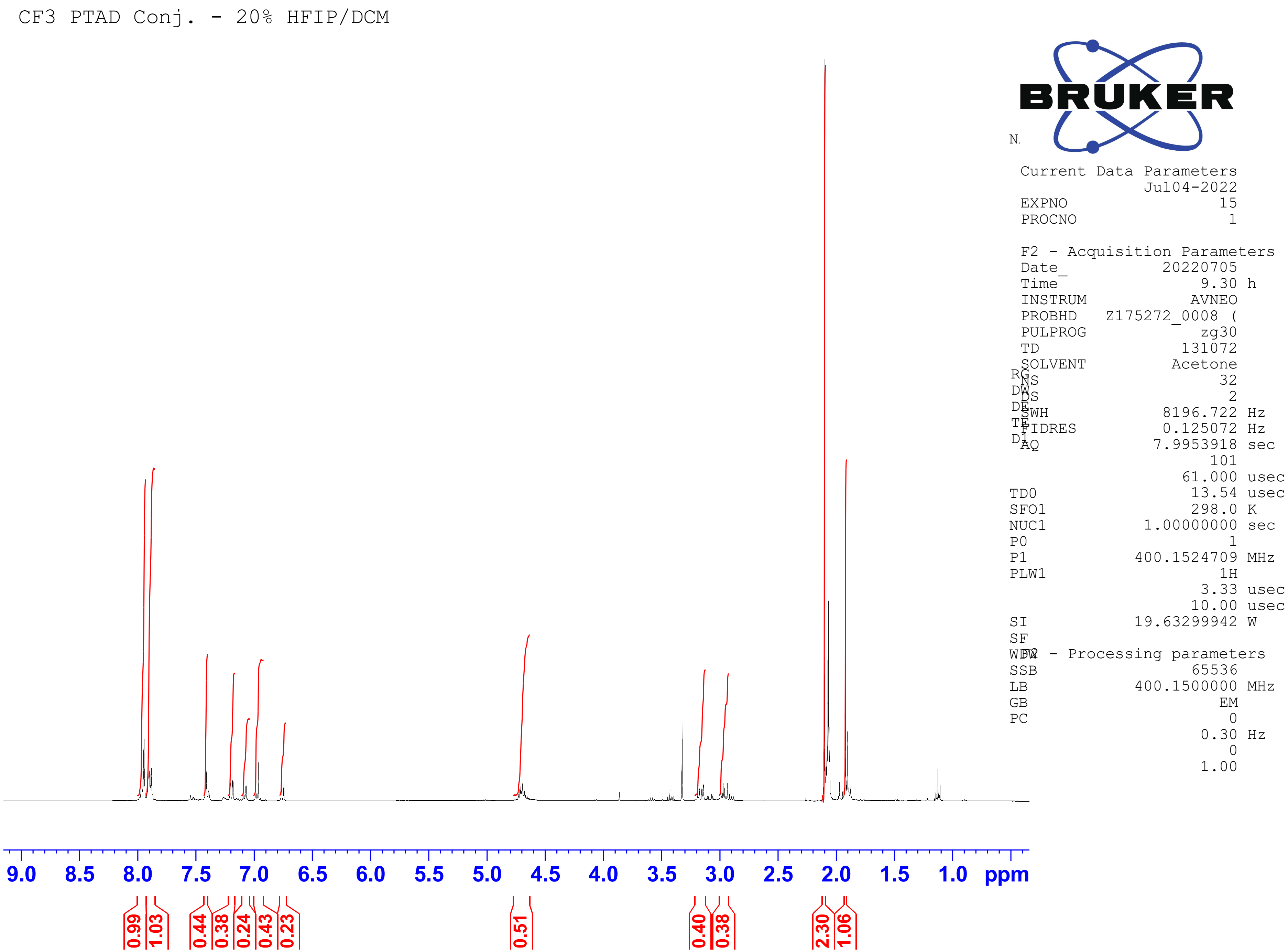

Figure S11. The ^{1}H NMR (400 MHz, $(CD_3)_2CO$) of compound **6b**

CF3 PTAD Conj. HFIP/DCM - Crude

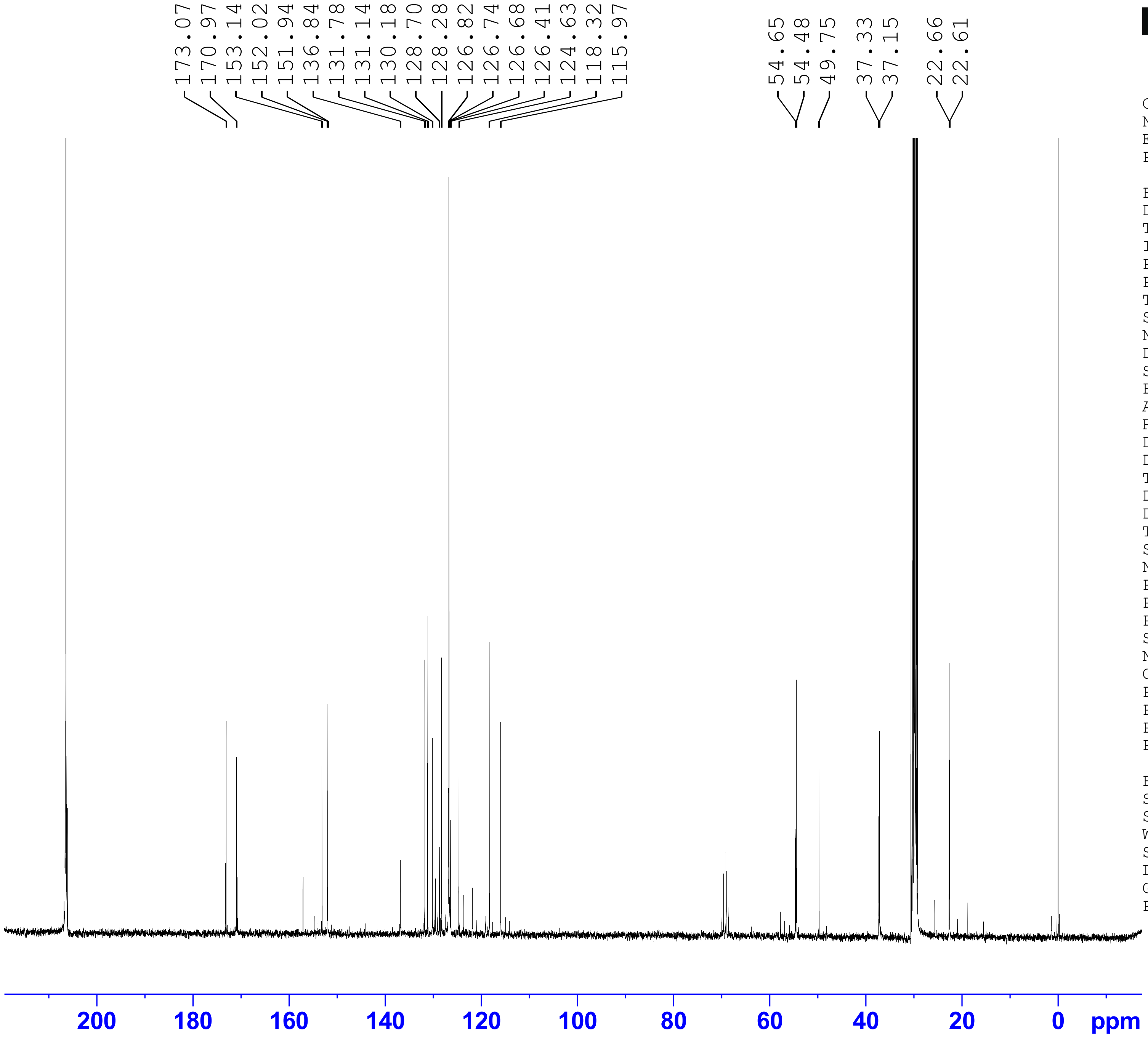

Current Data Parameters
NAME Jun30-2022
EXPNO 8
PROCNO 1

F2 - Acquisition Parameters
Date_ 20220702
Time 7.26 h
INSTRUM AVNEO
PROBHD Z175272_0008 (
PULPROG zgpg30
TD 131072
SOLVENT Acetone
NS 3600
DS 4
SWH 23809.523 Hz
FIDRES 0.363304 Hz
AQ 2.7525120 sec
RG 101
DW 21.000 usec
DE 6.50 usec
TE 298.0 K
D1 2.00000000 sec
D11 0.03000000 sec
TD0 1
SFO1 100.6278593 MHz
NUC1 13C
P0 3.33 usec
P1 10.00 usec
PLW1 58.38199997 W
SFO2 400.1516006 MHz
NUC2 1H
CPDPRG[2 waltz65
PCPD2 90.00 usec
PLW2 19.63299942 W
PLW12 0.24237999 W
PLW13 0.12192000 W

F2 - Processing parameters
SI 32768
SF 100.6177078 MHz
WDW EM
SSB 0
LB 1.00 Hz
GB 0
PC 1.40

Figure S12. The ^{13}C NMR (100.6 MHz, $(CD_3)_2CO$) of compound **6b**

CF3 PTAD Conj. - 20% HFIP/DCM

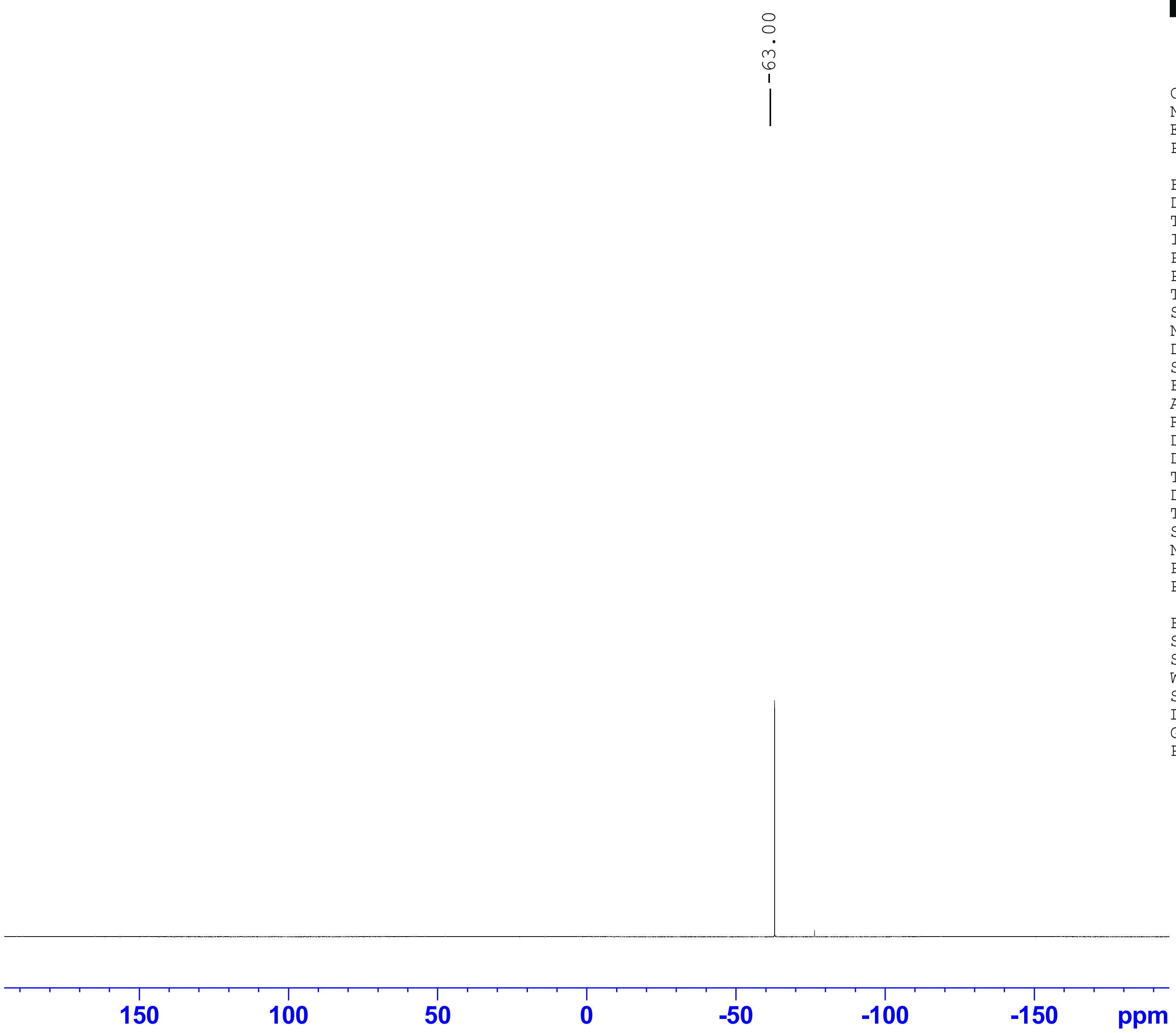

Current Data Parameters

NAME	Jul04-2022
EXPNO	17
PROCNO	1

F2 - Acquisition Parameters

Date_	20220706
Time	5.11 h
INSTRUM	AVNEO
PROBHD	Z175272_0008 (
PULPROG	zg
TD	131072
SOLVENT	Acetone
NS	128
DS	4
SWH	147058.828 Hz
FIDRES	2.243940 Hz
AQ	0.4456448 sec
RG	101
DW	3.400 usec
DE	6.50 usec
TE	298.0 K
D1	1.00000000 sec
TD0	1
SFO1	376.5171850 MHz
NUC1	19F
P1	12.00 usec
PLW1	45.00000000 W

F2 - Processing parameters

SI	65536
SF	376.5171850 MHz
WDW	EM
SSB	0
LB	0.30 Hz
GB	0
PC	1.00

Figure S13 The ^{19}F NMR (376.5 MHz, $(CD_3)_2CO$) of compound **6b**.

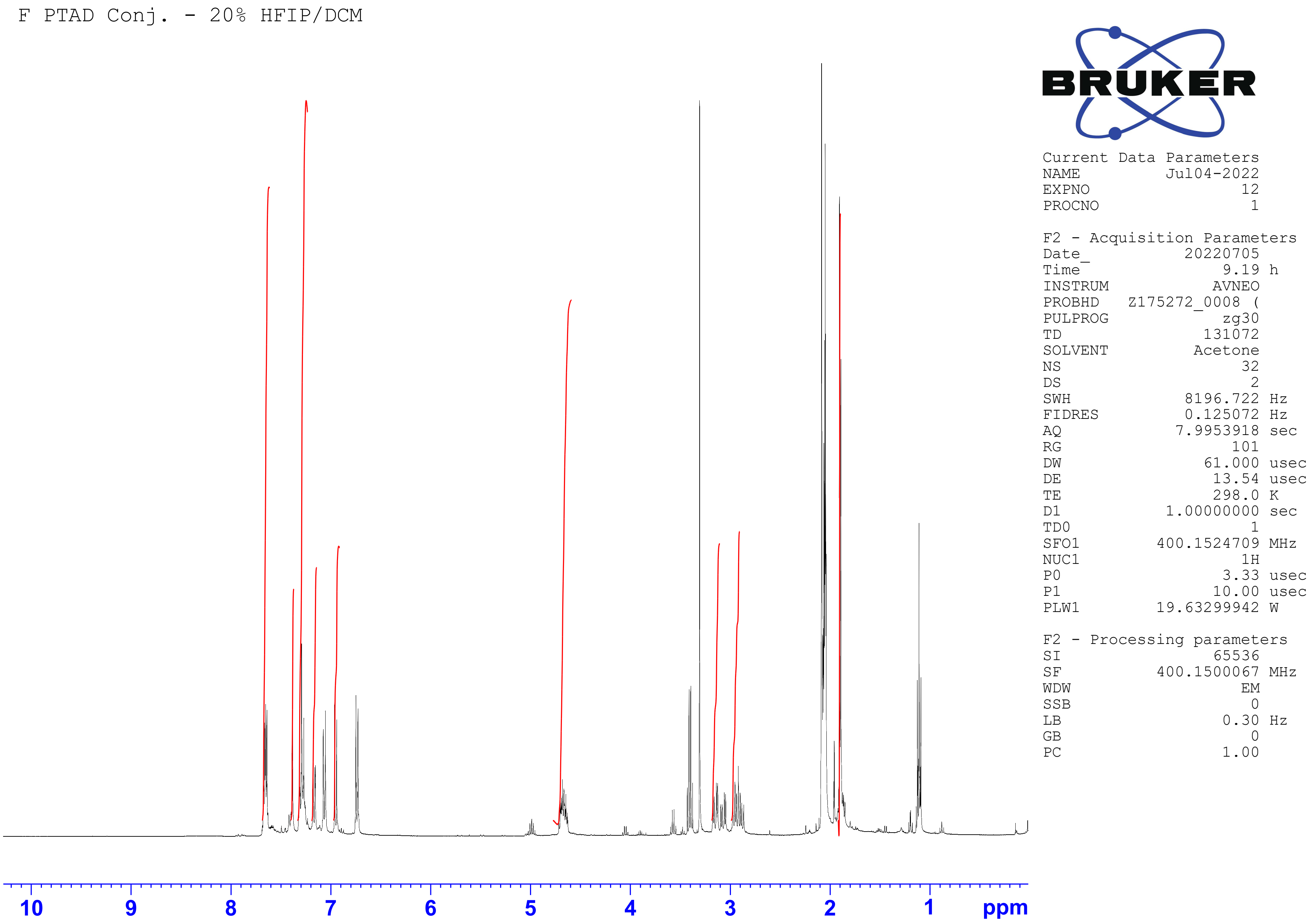

Figure S14. The ^{1}H NMR (400 MHz, $(CD_3)_2CO$) of compound **6c**

F PTAD Conj.

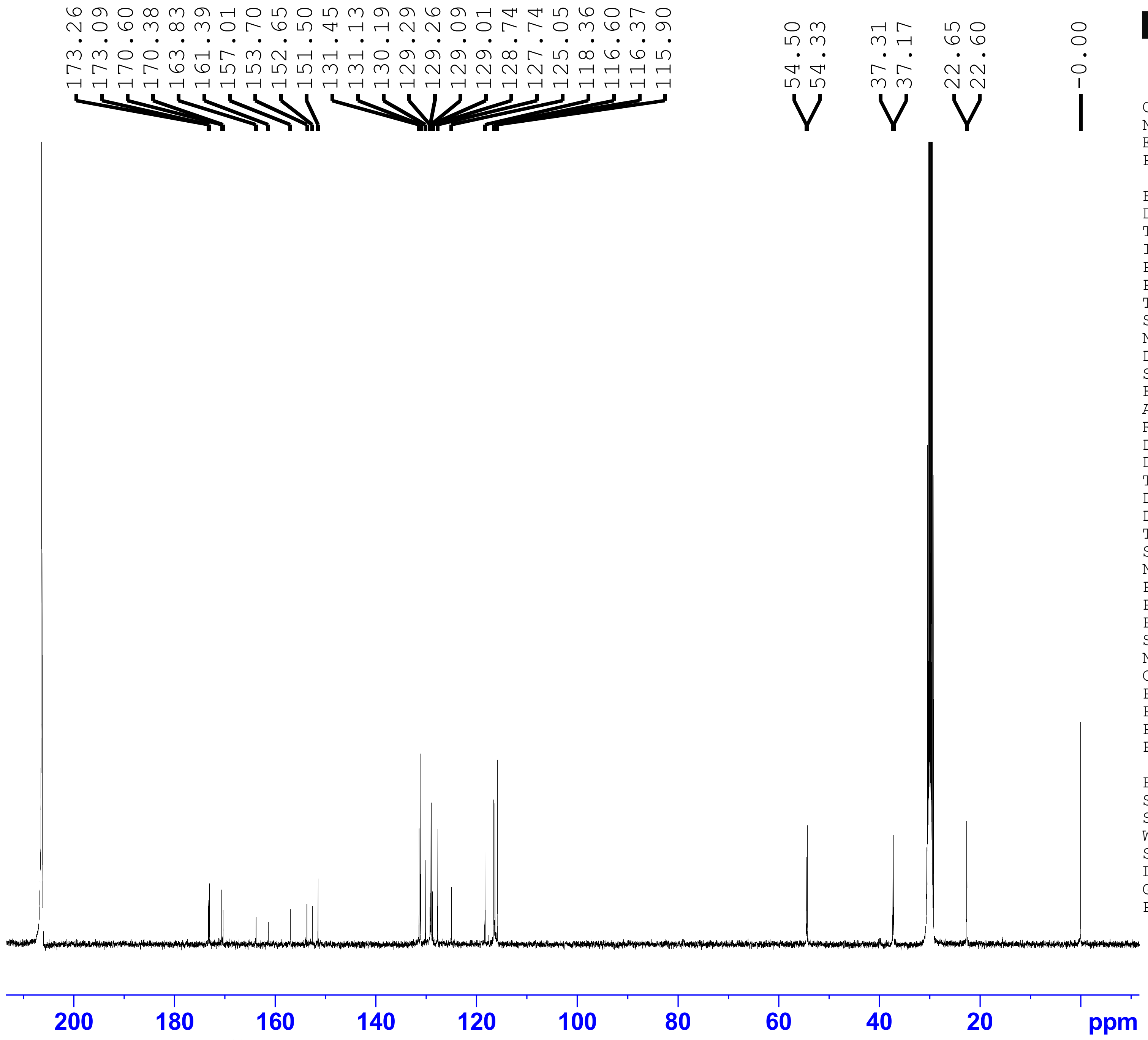

Current Data Parameters
NAME Aug22-2022
EXPNO 2
PROCNO 1

F2 - Acquisition Parameters
Date_ 20220823
Time 3.41 h
INSTRUM AVNEO
PROBHD Z175272_0008 (
PULPROG zgpg30
TD 65536
SOLVENT H2O+D2O
NS 3000
DS 4
SWH 23809.523 Hz
FIDRES 0.726609 Hz
AQ 1.3762560 sec
RG 101
DW 21.000 usec
DE 6.50 usec
TE 298.0 K
D1 2.00000000 sec
D11 0.03000000 sec
TD0 1
SFO1 100.6278593 MHz
NUC1 13C
P0 3.33 usec
P1 10.00 usec
PLW1 58.38199997 W
SFO2 400.1516006 MHz
NUC2 1H
CPDPRG[2 waltz65
PCPD2 90.00 usec
PLW2 19.63299942 W
PLW12 0.24237999 W
PLW13 0.12192000 W

F2 - Processing parameters
SI 32768
SF 100.6179757 MHz
WDW EM
SSB 0
LB 1.00 Hz
GB 0
PC 1.40

Figure S15. The ^{13}C NMR (100.6 MHz, $(CD_3)_2CO$) of compound **6c**

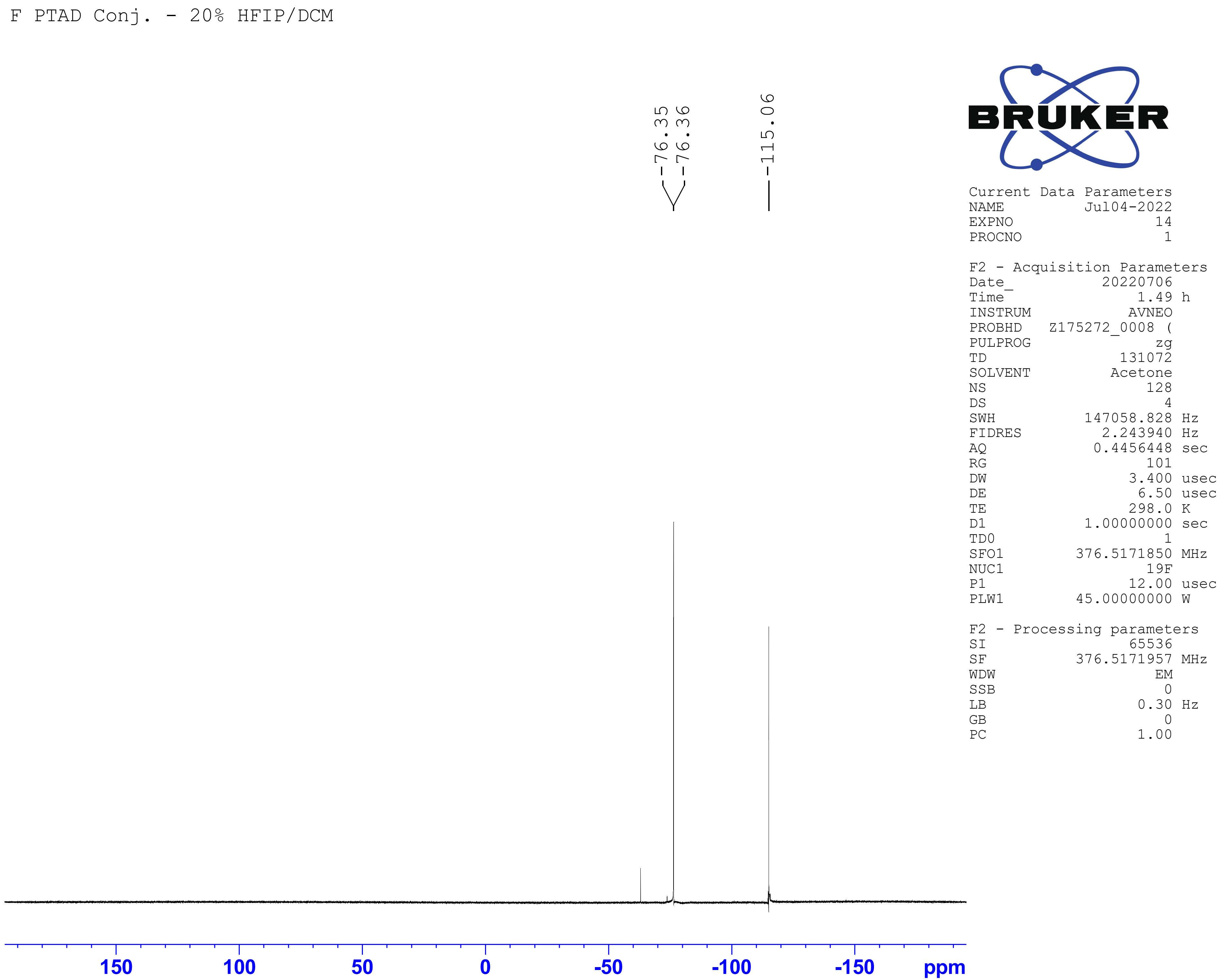

Figure S16. The ^{19}F NMR (376.5 MHz, $(CD_3)_2CO$) of compound **6c**

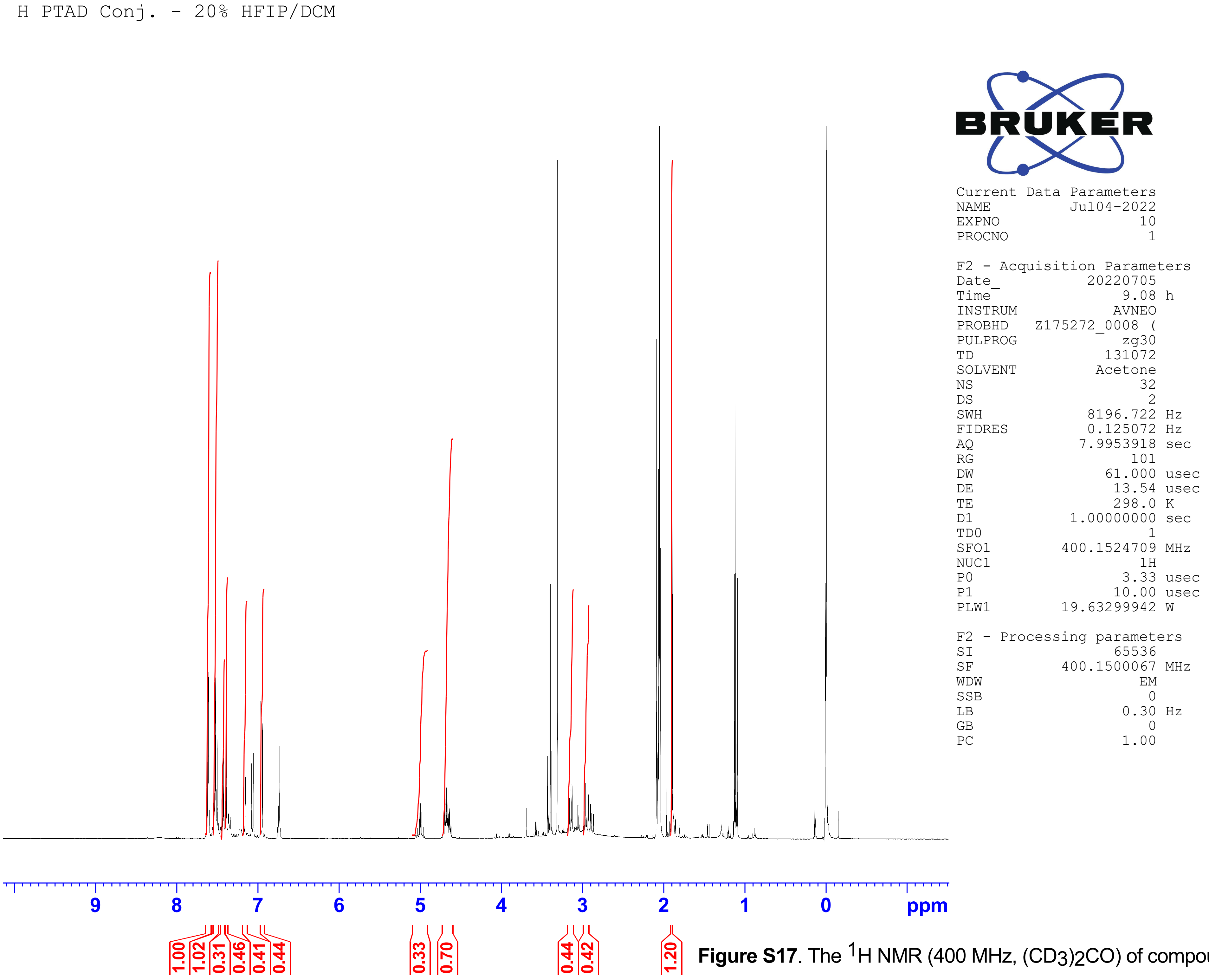

Figure S17. The ^{1}H NMR (400 MHz, $(CD_3)_2CO$) of compound **6d**

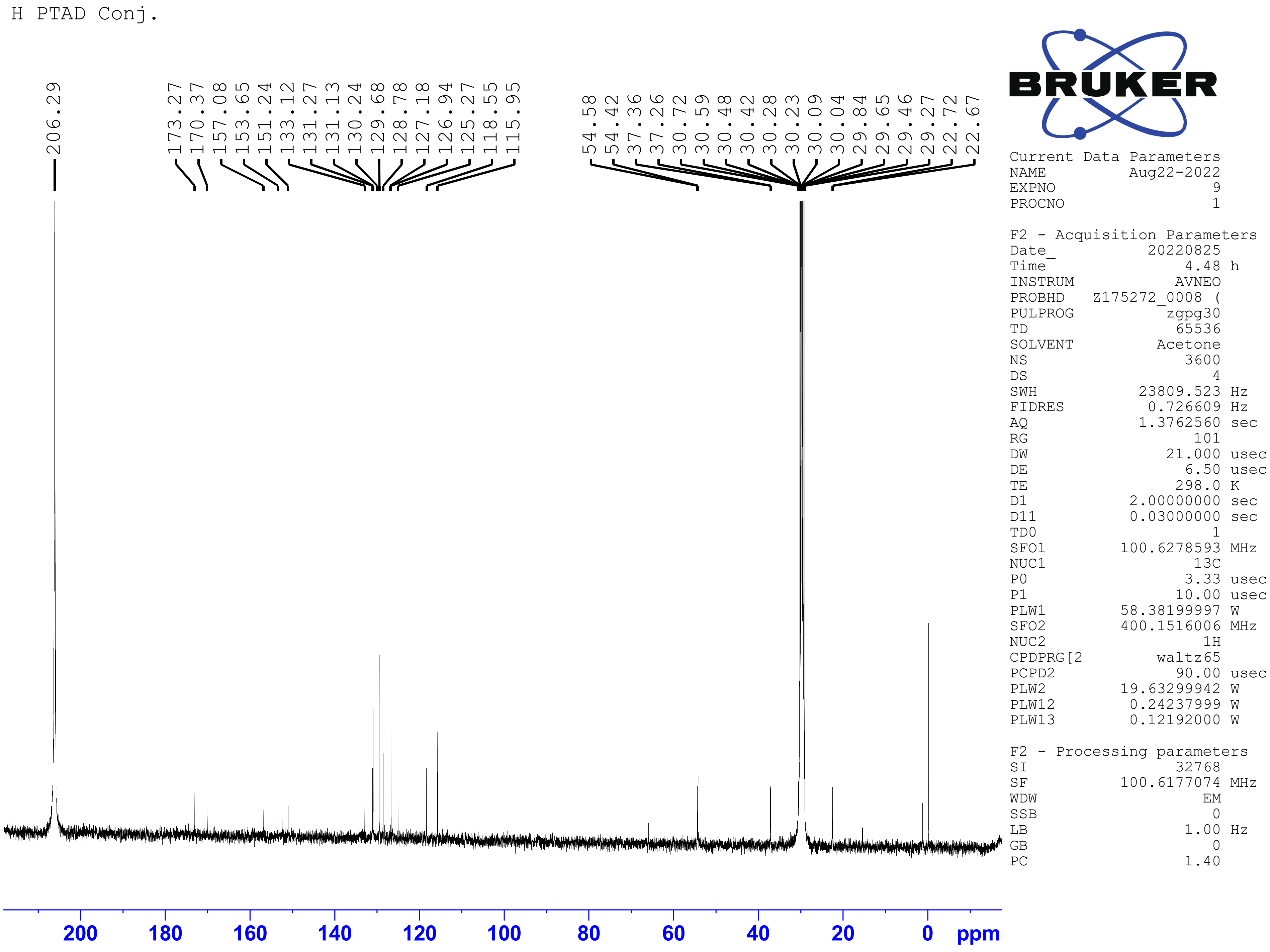

Figure S18. The ^{13}C NMR (100.6 MHz, $(CD_3)_2CO$) of compound **6d**

CH3 PTAD Conj. - 20% HFIP/DCM

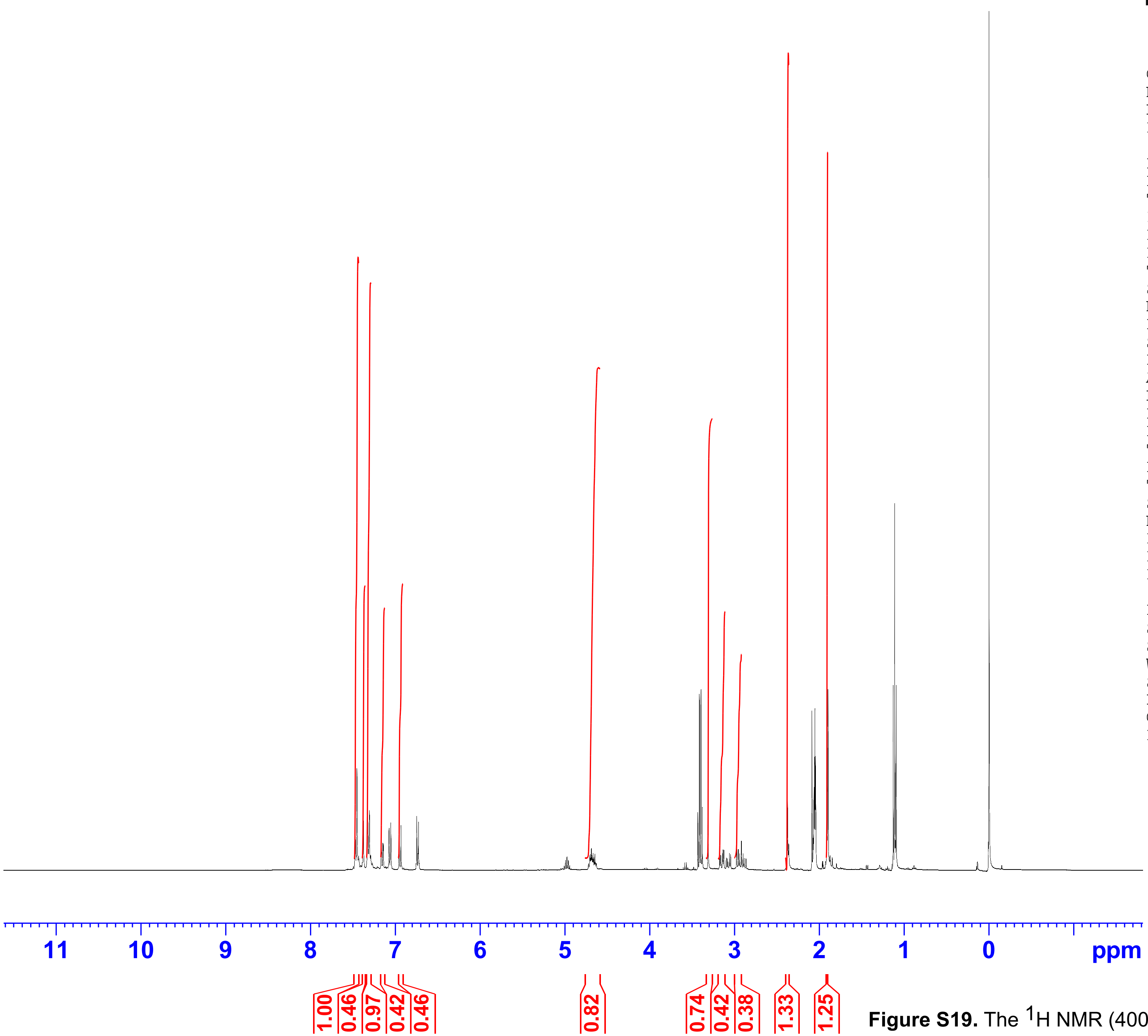

Current Data Parameters
NAME Jul04-2022
EXPNO 8
PROCNO 1

F2 - Acquisition Parameters
Date_ 20220705
Time 19.13 h
INSTRUM AVNEO
PROBHD Z175272_0008 (
PULPROG zg30
TD 131072
SOLVENT Acetone
NS 32
DS 2
SWH 8196.722 Hz
FIDRES 0.125072 Hz
AQ 7.9953918 sec
RG 71.8
DW 61.000 usec
DE 13.54 usec
TE 298.0 K
D1 1.00000000 sec
TD0 1
SFO1 400.1524709 MHz
NUC1 1H
P0 3.33 usec
P1 10.00 usec
PLW1 19.63299942 W

F2 - Processing parameters
SI 65536
SF 400.1500067 MHz
WDW EM
SSB 0
LB 0.30 Hz
GB 0
PC 1.00

Figure S19. The ^{1}H NMR (400 MHz, $(CD_3)_2CO$) of compound **6e**

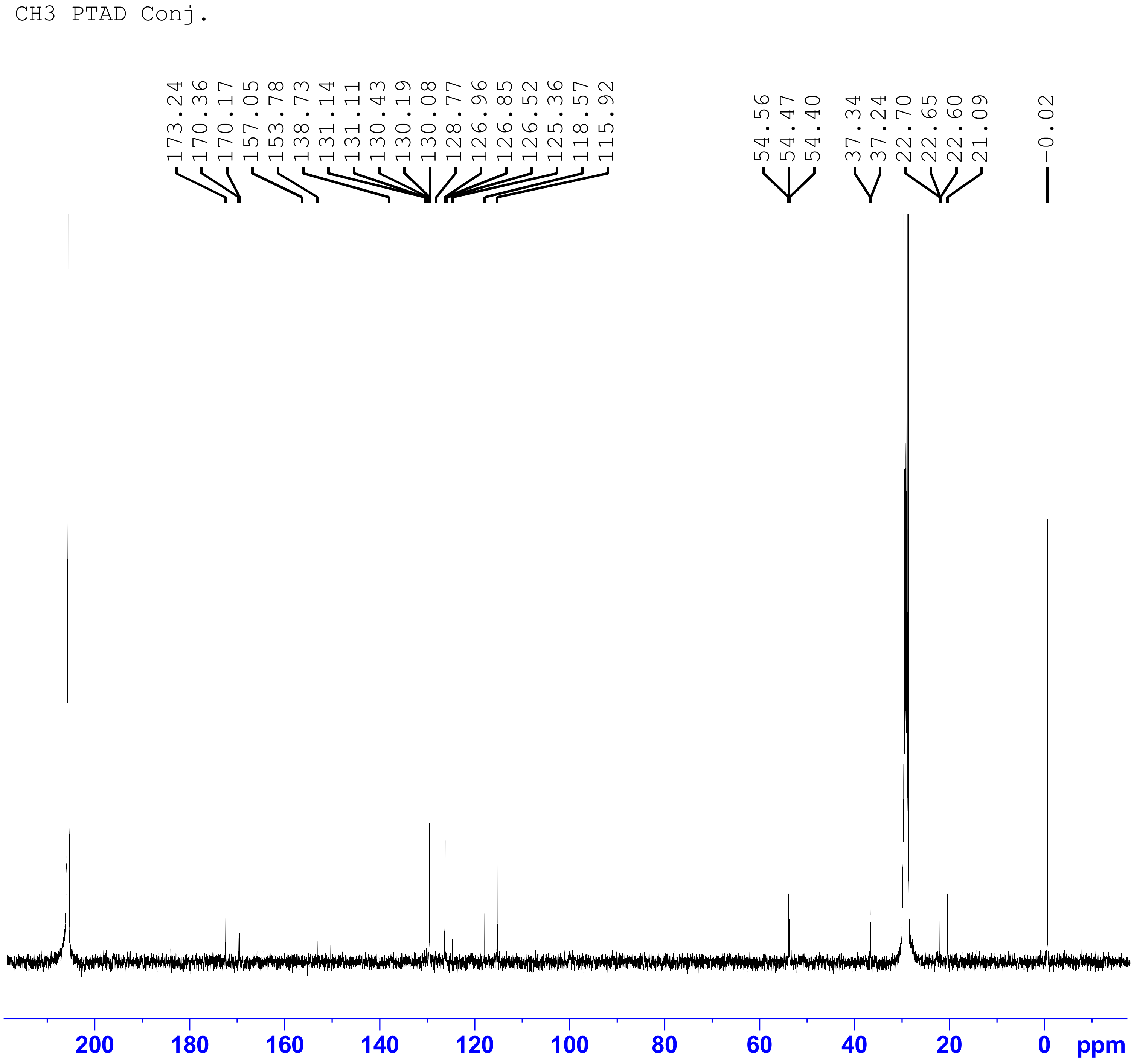

Current Data Parameters
NAME Aug22-2022
EXPNO 4
PROCNO 1

F2 - Acquisition Parameters
Date_ 20220823
Time 21.59 h
INSTRUM AVNEO
PROBHD Z175272_0008 (
PULPROG zgpg30
TD 65536
SOLVENT Acetone
NS 3000
DS 4
SWH 23809.523 Hz
FIDRES 0.726609 Hz
AQ 1.3762560 sec
RG 101
DW 21.000 usec
DE 6.50 usec
TE 298.0 K
D1 2.00000000 sec
D11 0.03000000 sec
TD0 1
SFO1 100.6278593 MHz
NUC1 13C
P0 3.33 usec
P1 10.00 usec
PLW1 58.38199997 W
SFO2 400.1516006 MHz
NUC2 1H
CPDPRG[2 waltz65
PCPD2 90.00 usec
PLW2 19.63299942 W
PLW12 0.24237999 W
PLW13 0.12192000 W

F2 - Processing parameters
SI 32768
SF 100.6177093 MHz
WDW EM
SSB 0
LB 1.00 Hz
GB 0
PC 1.40

Figure S20. The ^{13}C NMR (100.6 MHz, $(CD_3)_2CO$) of compound **6e**

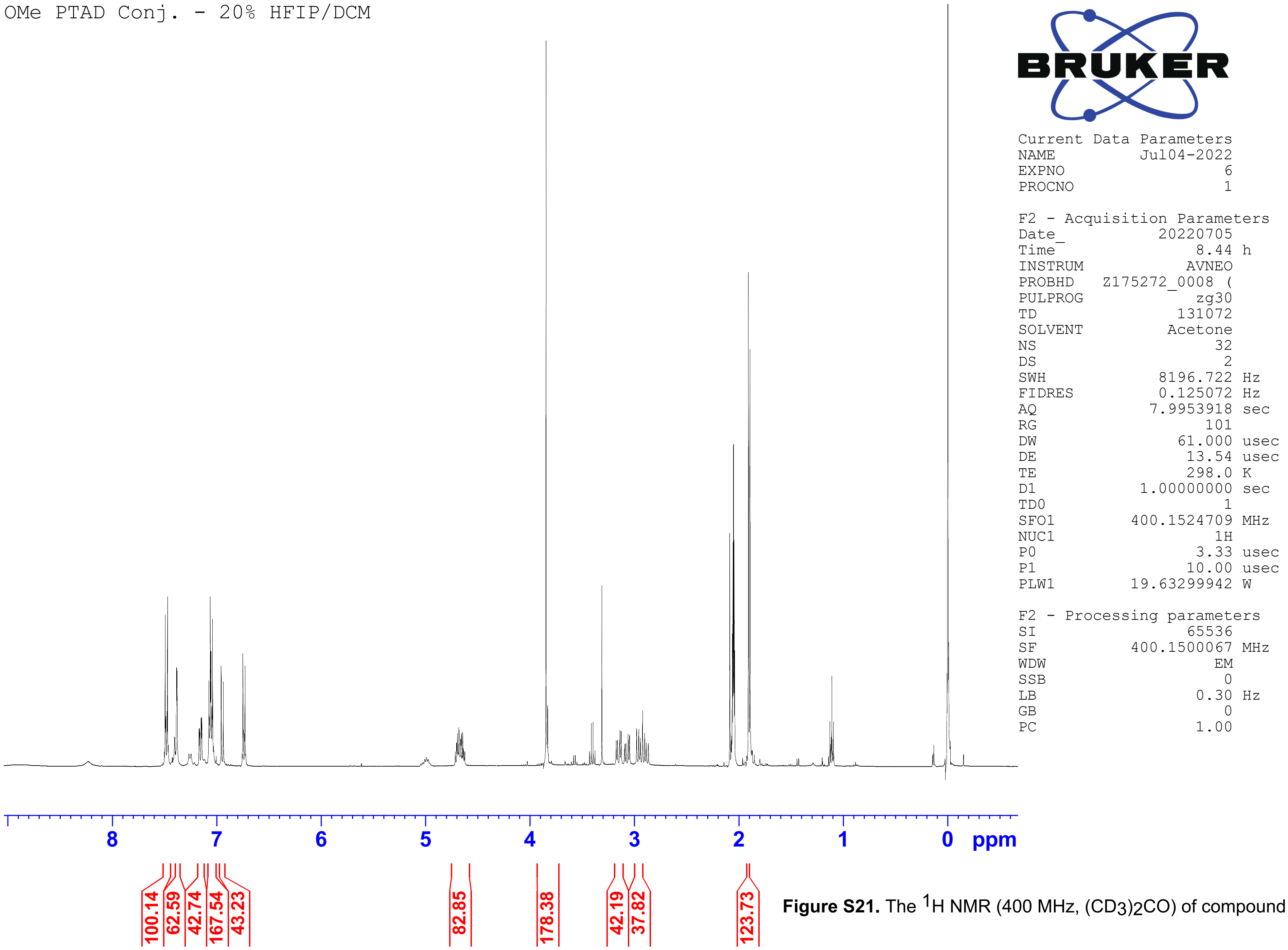

Figure S21. The ^{1}H NMR (400 MHz, $(CD_3)_2CO$) of compound **6f**

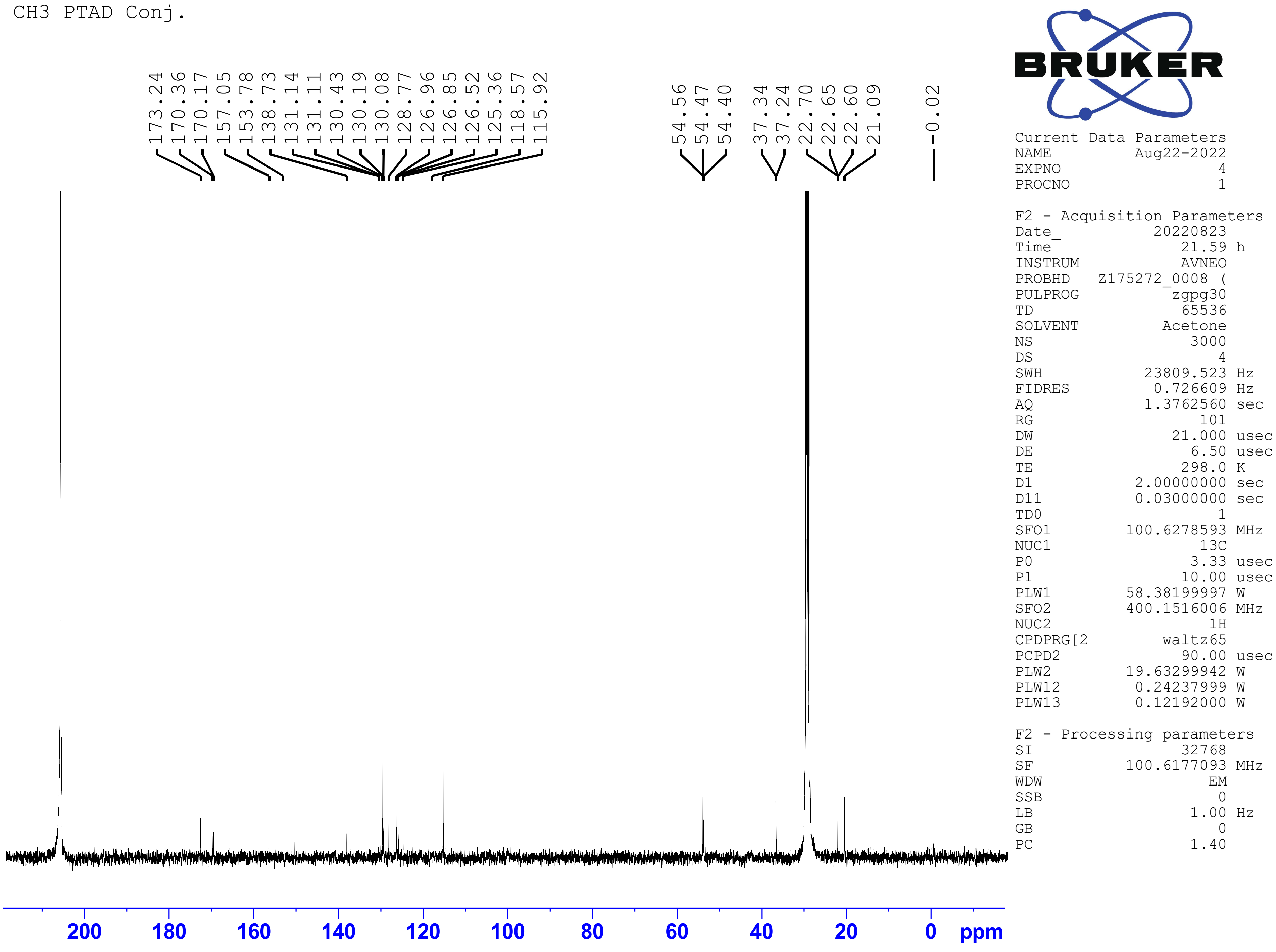

Figure S22. The ^{13}C NMR (100.6 MHz, $(CD_3)_2CO$) of compound **6e**
